# Medical Opinion and Movement

**Published:** 1907-01-26

**Authors:** 


### Medical Opinion and Movement.

Dr. A. S. Arkle, of Liverpool, has lately announced the results of an exhaustive examination of the children in three large Council schools in that city. He divides the schools into three classes —"A," "B," and "C." "A" schools, of children from comfortable homes; "B" schools, of children of the labouring classes; "C" schools, of children of the poorest class. He also examined children in the secondary schools for well-to-do people. The differences in height and weight of boys at the same age of the different classes are remarkable. At the age of seven there is a difference of 3.8 in. between the average height in the secondary schools and in the "C" schools. At eleven this difference has risen to 5.75 in., and at fourteen it amounts to 6.5 in. Schools "A" and "B" show intermediate proportionate differences. Similar differences appear in the weights. At seven years the most extreme difference amounts to 6.3 lb., and at fourteen it is 1 st. 9.25 lb. In other words, a secondary schoolboy at eleven is physically equal to a "C" boy at fourteen. As Dr. Arkle says, such differences in physique are of alarming proportions, and unless the children are helped in some way to get proper food and develop physically, the money spent on their mental education might as well be thrown into the sea.

The report just issued on the health of the Navy for the year 1905 is highly satisfactory. Of 111,020 men, the total number of cases of disease and injury was 81,568. This gives a decrease of 119.29 per 1,000 as compared with the eight years' average. The death-rate was 3.9 per 1,000 men, being a decrease of 1.42 on the same average. The Mediterranean station shows the highest invaliding ratio, and the East Indies the highest death-rate, the latter amounting to 9.14 per 1,000. In a memorandum on "Physical Culture and its Pitfalls" Staff-Surgeon A. Gaskell makes an interesting comparison between the muscularly strong man and the healthy man, and points out the dangers of excessive muscular development. Among the pitfalls of over-training are straining of the heart and circulatory system leading to valvular incompetence, atheroma, and aneurysm, and straining of the respiratory system causing emphysema and fibrosis of the lungs. The Swedish system has done much to emphasise the importance of developing quick co-ordinate movements of muscles without strain or excessive exertion, rather than the acquiring of bulky muscles of great lifting power, but of little practical utility. Staff-Surgeon Gaskell is of opinion that physical culture should be adapted to the man's avocation, and he suggests that a British system of physical training might be devised by analysing kinematograms of the movements of champions in each line of useful physical exercise.

Dr. W. J. Morton's report of the results of his experimental treatment of cancer cases with trypsin is now to hand. An analysis of these results shows that they are sufficiently favourable to encourage further investigation. The length of time during which the treatment has been tried is not sufficient to justify any definite statement of cure even in those cases where an apparent cure has been effected, but even on the most sanguine view of the cases one fails to find any justification for the enthusiasm with which the so-called "cure" was proclaimed by Dr. Saleeby in the lay press. The report includes altogether 29 cases, but three of them were sarcoma and two lupus, leaving 24 cases of cancer. Of these 11 cases, or nearly 50 per cent., consisted of epitheliomatous ulceration about the nose, cheek, and inner canthus of the eye. Although not so stated, the site of these growths strongly suggests rodent ulcer. It is against two of these cases only that we find the statement "cured up to date," and the most marked improvements also fall to these cases. Of the remaining 13 cases four died, four showed signs of general improvement in health, but in three cases only is definite local improvement noted. In two cases operation was resorted to. Many of the cases had already shown marked improvement under *x*-ray treatment. Dr. Morton is convinced that these trypsin injections exert a definite influence upon the malignant growth and also upon the general cachexia of the patient, but he reserves for the present an expression of opinion upon its permanent therapeutic value.

The Dublin authorities are bestirring themselves to adopt measures to stay the havoc caused by tuberculosis in the community. At a recent meeting of the Corporation it was resolved unanimously to make application to the Local Government Board with a view to forming the sanitary authorities of the city and county of Dublin into a united district for the purpose of erecting and maintaining a sanatorium for consumptives. It is to be regretted that some of the districts have withheld their consent to join the combination. In the meantime our lay contemporary, *The Freeman's Journal*, is assisting the movement by opening its columns to a discussion of the question, and is endeavouring to educate public opinion by publishing some very instructive letters on the subject from Sir Charles Cameron and other leading medical authorities. A comparison of the death-rates for the ten years ending 1880, with those for the same length of period ending 1904, is sufficient to convince anyone of the necessity for preventive measures against the disease. Whereas the deaths from the principal zymotic diseases have fallen from 45.2 per 10,000 persons living for the first period to 26.4 per 10,000 for the second, deaths from phthisis have risen from 29 to 32.9 per 10,000 persons living. The erection of a sanatorium is perhaps one of the first steps in the right direction, but much besides is required to be done. The people must be educated to regard consumption as an infectious and preventable disease, and must be taught methods of prophylaxis and disinfection. We would commend to the attention of the Dublin authorities the admirable dispensary system adopted in Edinburgh, of which a detailed description has recently appeared in our columns.

